# Low molecular weight heparins in COVID-19 patients: beware of augmented renal clearance!

**DOI:** 10.1186/s13054-020-03058-3

**Published:** 2020-06-10

**Authors:** Teresa Maria Tomasa-Irriguible, Sergi Martínez-Vega, Ester Mor-Marco, Alba Herraiz-Ruiz, Laura Raguer-Pardo, Carlos Cubells-Larrosa

**Affiliations:** 1 Intensive Care Medicine Department, Germans Trias i Pujol Hospital, Carretera del Canyet s/n, 08916 Badalona, Barcelona, Spain; 2 Anesthesiology Department, Germans Trias i Pujol Hospital, Badalona, Barcelona, Spain

Progressive respiratory failure is the primary cause of death in the coronavirus disease 2019 pandemic (COVID-19). Histologic analysis of pulmonary vessels in patients with COVID-19 has shown widespread thrombosis with alveolar capillary microthrombi [1]. COVID-19 is known to be associated with thrombotic phenomena that are related to the high mortality of this disease and proper treatment can improve the survival of these patients [2–5].

Augmented renal clearance (ARC) is a phenomenon that frequently occurs in critically ill patients and can cause therapeutic failure of renal removal drugs [6].

Here is presented a clinical case to illustrate the topic. A 52-year-old man without drug allergies or toxic habits, with a history of dyslipidemia and obesity (BMI 32), was admitted to the hospital on 19 April with bilateral COVID-19 pneumonia. D-dimer value was 422 ng/mL at ICU admission. He needed 3 sessions of prone position with a good response, so that on hospital day 12, intubation was removed. On hospital day 14, he was discharged from the ICU to the ward. However, that afternoon, in the context of postural change, he developed oxygen desaturation at 55% with chest pain. D-dimer was 30,000 ng/mL. Electrocardiogram showed sinus tachycardia 130 bpm with typical S1Q3T3. An echocardiogram showed severe right cavity dysfunction, suggesting pulmonary thromboembolism (PE). An arteriography was performed confirming the suspicion and mechanical thrombectomy was performed. Cavography was also performed observing parietal thrombus in the infrarenal inferior cava and the ipsilateral iliac, thus a vena cava inferior filter was implanted.

Afterwards, the administered deep vein thrombosis prophylaxis (DVTP) regimen was reviewed. He had received enoxaparin 60 mg daily since admission. Hospital protocol advises 40 mg for patients below 80 kg and 60 mg for patients above 80 kg. The patient weighed 80 kg, so he was given the dose he was supposed to receive. The renal function of the patient was also reviewed. He always had a glomerular filtration rate (GFR) above 90 mL/min/1.73 m^2^, estimated by the Chronic Kidney Disease Epidemiology Collaboration (CKD-EPI) formula. Nevertheless, once a week in ICU, we collect 24-h urine, so as to be able to calculate the real GFR, and we found that he had ARC of up to 196 mL/min/1.73 m^2^ on hospital day 10.

Is it possible that the prophylactic dose of enoxaparin 60 mg daily was insufficient to prevent deep vein thrombosis (DVT) in this patient with COVID-19 plus ARC? Is it also possible that a two-fold increase in the GFR may also double enoxaparin removal? If so, could the same thing have happened to others? And what about the therapeutic anticoagulant dosage?

Subsequently, we analyzed the incidence of ARC in ICU patients during the COVID-19 pandemic. Of the 47 patients admitted to the ICU for COVID-19, 18 (38.3%) had ARC. Compared to non-ARC’s, both DVT and PE were higher in ARC (44% vs. 31%) and (33% vs. 10%, *P* = 0.025), respectively. We also analyzed 2 patients with DVT plus ARC who were receiving 150 mg daily of enoxaparin (1.5 mg/kg/day) and the antiXa activity was 0.27 and 0.28 UI/mL, respectively, when the effective range comprises 0.4–1.1 UI/mL.

Regarding the COVID-19 pandemic and the need of anticoagulants for DVTP, physicians have to be aware of this ARC phenomenon and the risk of under exposure to low molecular weight heparins. This letter is to alert physicians as to the high incidence of ARC in these patients and provide warning regarding the fact that regular doses of low molecular weight heparin might not be protective enough for DVT and PE in this type of COVID-19 patient.

## Data Availability

Not applicable

## Abbreviations

ARC: Augmented renal clearance
BMI: Body mass index
CKD-EPI: Chronic Kidney Disease Epidemiology Collaboration formula
COVID-19: Coronavirus disease 2019 pandemic
DVT: Deep vein thrombosis
DVTP: Deep vein thrombosis prophylaxis
GFR: Glomerular filtration rate
ICU: Intensive care unit
PE: Pulmonary thromboembolism
